# An Interventional Health Education Study to Transition the General Population’s Opinion on Organ Donation From Reluctance to Acceptance

**DOI:** 10.7759/cureus.75792

**Published:** 2024-12-16

**Authors:** Sree Harichandana K, Kiran B, Kala P, Jamuna Rani

**Affiliations:** 1 Pharmacology, SRM Medical College Hospital and Research Centre, Chennai, IND

**Keywords:** awareness, barrier, general population, organ donation, organ donor card

## Abstract

Introduction: Organ donation refers to the collection of a human organ from a living or deceased donor and its transplantation into a recipient. An organ transplant recipient is a patient with organ failure who will not survive unless he receives a new organ. Although the benefits of organ transplantation are undeniable, there is a significant gap between the number of donors and recipients, as the demand for organs greatly surpasses the available supply.

Methodology: This interventional health education study aimed to increase awareness regarding organ donation through a survey consisting of pre- and post-questionnaires. The study included 226 participants from the general population of Tamil Nadu, aged 18 years or older and of both genders. The study was conducted over three months, from September to November 2023.

Results: Of the 226 study participants, 220 (97.30%) belong to the 18-40 age group, and 154 (68.1%) were female participants. Before the educational intervention, 132 (58.40%) participants were aware of organ donation. After the educational intervention, knowledge about the organ donation card increased from 101 (44.69%) participants to 152 (67.25%), and the inclination to donate organs rose from 128 (56.63%) to 151 (66.81%). Overall, awareness about organ donation improved from 132 (58.40%) to 175 (77.43%). The study noted that 128 (56.63%) participants viewed religious beliefs as a barrier to organ donation, a perception that remained unchanged, with 152 (67.25%) participants still holding this view even after the educational intervention.

Conclusion: This interventional health education study aimed to inform the general population about the importance of organ donation in India, where road traffic accidents frequently lead to brain death, and a lack of awareness potentially limits organ donation opportunities. Despite high levels of education, many individuals remain reluctant to donate organs and lack an organ donor card. Although there is a strong willingness to donate, registration rates remain low, suggesting the presence of potential obstacles, such as religious beliefs and insufficient knowledge about organ donation. Implementing targeted educational campaigns that involve spiritual leaders and provide accurate data about organ donation is crucial. This could improve public awareness and participation. Implementing an opt-out system, wherein individuals are presumed willing to donate unless they explicitly decline, could significantly increase donor rates.

## Introduction

Organ transplantation represents an exceptional and noteworthy contribution to modern medicine, offering a life-saving solution for individuals suffering from end-stage organ failure. Organ donation refers to the collection of a human organ from a living or deceased donor and its transplantation into a recipient. An organ transplant recipient is a patient with organ failure who will not survive unless he receives a new organ. Organ transplants not only save lives but also substantially improve the quality of life for patients. For example, kidney recipients may no longer need dialysis, heart recipients can resume daily activities, and liver transplant recipients can regain their health and vitality. Organ donation transforms individuals from a state of severe illness and dependence on medical interventions to one of restored health and independence. It also offers a lasting opportunity to create a humanitarian legacy, allowing individuals to make a life-saving impact even after death. This act of donation provides profound emotional significance for both the donor's family and the recipients. The financial benefits of organ donations are substantial. Transplanted organs significantly reduce ongoing medical expenses associated with dialysis, medication, and other expensive treatments. Individuals in good health who receive organ transplants are often able to return to the workforce and make valuable contributions to society, consequently improving community health and economic well-being. The regulation of organ donation is stringent, guaranteeing that the process is ethical and transparent. This encompasses the necessity for donors to provide informed consent, and the process must characterized by fairness and equality. Several countries have established protocols designed to prioritize individuals based on their medical needs and compatibility. Organ donation also has a global impact. Some nations employ organ donation regulations that operate on the principle of presumed consent, where individuals are automatically considered organ donors unless they actively decide to opt out. In contrast, other nations adhere to systems that require prior authorization from individuals. The interconnected nature of healthcare and travel emphasizes organ donation as a global issue and ethical debate. Despite the clear benefits of organ transplantation, a significant gap remains between the number of donors and recipients, as the demand for organs greatly surpasses the available supply. In India, despite being second only to the United States in total transplants, the organ donation rate remains low at a meager 0.65 per million population [1]. According to the United Network for Organ Sharing database, the United States conducted 41,354 transplants in 2021. Despite over 6,500 living donor transplants and 13,800 deceased donations during that timeframe, the demand for donor organs far surpasses the availability [2]. According to the European Directorate for the Quality of Medicines and Health Care (EDQM), Spain leads with 47 deceased donors per million in 2022. The rates of organ donation in the United States, Portugal, France, and the United Kingdom were 44.5, 31.5, 24.8, and 20.6 donors per million worldwide, respectively [3]. India's organ donation rates consistently increased over the past decade, from 4990 in 2013 to 16,041 in 2022, with 13,338 living transplants and 941 deceased donor contributions. Tamil Nadu has the highest number of living organ transplants in India, with 1690 living organ transplants and 555 deceased donor transplants [4]. A comprehensive analysis of Indians, with the vast majority being Indians residing in India and the remaining participants being people of Indian descent residing in other countries, revealed favorable attitudes toward organ donation registration but a reduced willingness and even lower frequency of organ donor registration [4]. A study conducted in northeast India found that while 285 (79.17%) demonstrated full awareness of organ donation, only 216 (57%) expressed willingness to become organ donors, with 45 (12.5%) citing religious beliefs as a hindrance to organ donation [5]. Comparative studies involving medical students from Badnapur (46; 46.94%) [6], Tamil Nadu (57 (34.55%) male students, 64 (24.62%) female students) [7], Gujarat (153; 61.2%) [8], Hyderabad (95; 65.4%) [9], and Punjab (193; 96.5%) [10] revealed varying levels of knowledge and awareness, highlighting the urgent need to address organ donation hesitancy across the population. The disparity between awareness and action concerning organ donation is substantial, with obstacles such as myths, sociocultural attitudes, religious perspectives, and misconceptions substantially hindering the willingness to donate organs. Overcoming these obstacles is essential for increasing organ donation rates. Hence, this study aimed to assess awareness and barriers to organ donation among the general population of Chengalpattu District and to evaluate the impact of interventional health education in reducing these barriers, fostering positive attitudes toward organ donation, and increasing donor registrations to save lives.

## Materials and methods

Study design and setting

This interventional health education study aimed to increase awareness regarding organ donation through a survey consisting of pre- and post-questionnaires. This study included 226 participants from the general population of Tamil Nadu, aged 18 years or older and of both genders. The study was conducted over three months, from September to November 2023. Ethical approval was obtained from the institutional ethical committee (SRMIEC-ST0723-1483) before the study began. Written informed consent was obtained from all participants after providing complete information.

Study participants

The study included male and female residents of Tamil Nadu, aged 18 years or older. Those who voluntarily agreed to participate were included, and those who declined were excluded. Study participants were assured that their personal information would remain confidential.

Study instrument

A preexisting questionnaire, adapted from prior studies, was used to assess the level of awareness and barriers related to organ donation. A skilled translator converted the English version into Tamil, and the Tamil version was then translated into English to guarantee the precision of the questionnaire. The revised questionnaire was reviewed by 10 subject matter experts, with each item achieving a content validity ratio exceeding 0.99. The questionnaire underwent an initial testing phase involving 30 participants and confirmed that the questionnaire was clear and easily comprehensible. Demographic information of the participants was collected utilizing a unique case report form. The questionnaire consisted of two sections: one assessing awareness and the other assessing barriers related to organ donation. Both awareness and barrier sections comprised 10 closed-ended questions each.

Study procedure

The study commenced after receiving approval from both the scientific and institutional ethical committees. It included 226 participants residing in Tamil Nadu, aged 18 years and older and of both genders. The study participants were recruited using a convenient sampling approach, after obtaining signed informed consent. They were given a detailed explanation of the study protocol. Before the educational intervention was conducted, the validated questionnaire was distributed in the local dialect, with instructions to complete it offline. For the educated population, the questionnaire was sent via Google Forms. Following this, the subject matter expert supervised a series of 8-10 training sessions, each lasting 30 minutes. The sessions include group discussions, campaigns, community outreach programs, and posters, all carried out in the local dialect. Participants received extensive information on organ donation, its life-saving potential, the organs that can be donated, and the optimal timing for donation. They were also informed about brain death, legal regulations related to organ donation, and the organ donation card. They were later asked to complete the posttest using the same questionnaire, either online via Google Forms or offline, on the same day. Data obtained from the pre- and posttests were collected and analyzed.

Sample size calculation

Sample size calculation was performed using the OpenEpi Info Version 3 Web Tool (Dean AG, Sullivan KM, Soe MM. OpenEpi: Open Source Epidemiologic Statistics for Public Health, Version. www.OpenEpi.com, updated 2013/04/06). Assuming a population size (N) of 1,000,000 and a hypothesized frequency (p) of 15.6% at a 95% confidence interval and a design effect of 1, a sample size of 203 was calculated. To account for a 10% nonresponse rate, the final sample size was increased to 224.

Statistical analysis

The results were analyzed using descriptive statistics, which were presented as total numbers and percentages. A paired t-test was applied to compare the differences in awareness and barriers toward organ donation between the pretest and posttest. A p < 0.001 was considered statistically significant. Statistical analysis was conducted using JASP software, Version 19 (The JASP Team, Amsterdam, Netherlands).

## Results

A total of 226 participants were recruited for the study. Of these, 220 (97.30%) belong to the 18-40 age group and six (2.70%) belong to the 41-60 age group. There were 73 (32.3%) male and 154 (68.1%) female participants. In terms of education, 99 (43.80%) were undergraduate students, 58 (25.66%) had completed the 12th standard, and 59 (26.11%) were graduates. Occupation-wise, 157 (69.46%) were students, and 36 (15.92%) were professionals (Table 1).

**Table 1:** Demographic details of the study population

Age in years	N	%
18-40	220	97.30%
41-60	6	2.70%
Above 60	0	0
Gender		
Male	73	32.3
Female	154	68.1
Qualification		
12th pass	58	25.66
Undergraduate	99	43.80
Graduate	59	26.10
Doctoral	2	0.88
Postdoctoral	0	0
Other	8	3.53
Occupation		
Student	157	69.46
Professional	36	15.92
Businessman	12	5.30
Private employee	8	3.53
Government employee	8	3.53
Homemaker	4	1.76
Other	1	0.44

The pretest of the knowledge section revealed that only 132 (58.40%) participants were aware that organs can be donated to save lives and 112 (49.55%) were knowledgeable about living and deceased organ donation. A total of 127 (56.19%) participants demonstrated knowledge regarding the organs eligible for donation. Additionally, 123 (54.42%) were aware that parents or close relatives can consent to organ donation on behalf of minors. Meanwhile, 117 (51.76%) participants were aware that organs cannot be sold, and 140 (61.94%) recognized that a brain-dead patient is a potential donor. Regarding willingness to donate, 124 (54.86%) expressed a willingness to donate organs as next of kin, 101 (44.69%) were aware of organ donor cards, and 128 (56.63%) indicated a willingness to donate their organs. Following the educational intervention, the posttest results showed a statistically significant improvement in participants' knowledge, as shown in Table 2.

**Table 2:** Assessment of awareness about organ donation among the general population before and after the health education intervention (total study participants = 226) A paired t-test was used for comparison between the pretest and posttest. *p < 0.001 was considered statistically significant.

Organ donation awareness questionnaire	Study participants with correct responses			
	Pretest	Posttest	t-test value	p-value
1. Organs can be donated to save the lives of other people	132 (58.40%)	175 (77.43%)	7.271	<0.001*
2. Organs can be donated both during life and after death	112 (49.55)	161 (71.23%)	7.029	<0.001*
3. All the following organs can be donated: blood, kidney, liver, eye, skin, heart, lungs, pancreas, bone marrow, brain, sperm, egg, and embryo	127 (56.19%)	169 (74.77%)	6.781	
4. If the deceased donor is under 18 years old, one of the parents or any near relatives authorized by the parents will give the permission to donate the organs	123 (54.42%)	148 (65.48%)	3.519	<0.001*
5. Is it possible to sell organs?	117 (51.76%)	145 (64.15%)	4.898	
6. A person on artificial life support declared as brain-dead can be a potential organ donor	140 (61.94%)	162 (71.68%)	3.772	<0 .001*
7. As the next of kin, would you be willing to donate the organ(s) of a brain-dead relative and save the lives of people who need it crucially?	124 (54.86%)	159 (69.02%)	6.421	<0 .001*
8. Indian Law recognizes brain death as a form of death, so that organs can be donated to patients in need	126 (55.75%)	153 (67.69%)	4.168	
9. Do you know about an organ donor card?	101 (44.69%)	152 (67.25%)	6.958	
10. Are you willing to register yourself as an organ donor and always carry an organ donor card with you to donate your organs after your death?	128 (56.63%)	151 (66.81%)	3.898	<0 .001*

Among the study participants, 123 (54.42%) were unaware of organ donation, and 130 (57.52%) believed that brain death should be declared by four expert doctors, considering the process time-consuming. A delay in declaring brain death was identified as the most significant challenge to organ donation by 143 (63.27%) respondents. Additionally, 121 (53.53%) respondents supported the necessity for the transplant coordinator to consult the family of the brain-dead individual without delay. A total of 147 (65.04%) respondents expressed the opinion that family members were often not convinced regarding organ donation in cases of brain death. Furthermore, 116 (51.32%) participants reported that families of brain-dead individuals often decline organ donation due to hopes for recovery. According to 118 (52.21%) respondents, family members may change their minds regarding organ donation. A delay in persuading the family was also found to increase the risk of organ deterioration, as reported by 112 (49.6%) participants (Table 3). However, following the educational intervention, there was a statistically significant improvement in the participants' attitudes toward organ donation.

**Table 3:** Assessment of barriers to organ donation among the general population before and after the health education intervention (total participants = 226) A paired t-test was used for comparison between the pretest and posttest. *p < 0.001 was considered statistically significant.

Barriers to organ donation questionnaire	Study participants with correct responses			
	Pretest	Posttest	t-test value	p-value
1. There is a lack of awareness about organ donation among people	123 (54.42%)	153 (67.69%)	5.868	
2. A person’s death has to be certified by a board of four medical experts before the person’s organs can be surgically removed, which is cumbersome and time-consuming in India	130 (57.52%)	162 (71.68%)	6.092	
3. The delay in declaring brain death or brain death diagnosis is a barrier to organ donation	143 (63.27%)	180 (79.64%)	6.637	<0.0018*
4. The transplant coordinator of the hospital should approach the family regarding organ donation immediately after identifying the brain-dead or deceased donor, without much delay	121 (53.53%)	146 (64.60%)	3.592	
5. Inability of the clinicians to convince the family to donate organs when the person is deceased or brain-dead?	147 (65.04%)	160 (70.79%)	3.706	
6. Hopes for recovery of the brain-dead make the next of kin refuse organ donation	116 (51.32%)	150 (66.37%)	3.483	<0.001*
7. The family may or may not agree and can, until the last minute before retrieval, change their minds about organ donation, which is the biggest issue	118 (52.21%)	158 (69.91%)	6.956	<0.001*
8. Do you think the longer it takes to retrieve the organs, the more they will deteriorate inside the body?	112 (49.55%)	149 (65.92%)	3.465	
9. Lack of government's proper initiative to educate people regarding organ donation	109 (48.23%)	143 (63.27%)	4.579	
10. Are there any racial, ethnic, and/or religious perspectives that prevent the donor from organ donation	128 (56.63%)	152 (67.25%)	3.898	<0.001*

## Discussion

Unlike other studies conducted among medical or nursing students, or other healthcare professionals, this study is one of the few focusing on the general population in Chengalpattu District, Tamil Nadu, to assess awareness and barriers related to organ donation. This study highlighted a notable level of public awareness about the importance of organ donation. Prior to the educational intervention, 132 (58.40%) participants knew about organ donation, which was comparable to the results of a study among postgraduate students and interns in Karnataka, where all participants were aware of organ donation and 87 (71.3%) were willing to donate organs. However, the participants expressed concerns about the lack of organ donation topics in the curriculum [11]. In contrast, awareness was significantly higher (175; 87.5%) among commerce students in Ahmedabad, compared to the current study [12]. An organ donor card provides a simple way for individuals to express their intent to donate their organs posthumously. Although it is important, discussing organ donation preferences with family members is also crucial to ensure awareness and support. In this study, following the educational intervention, awareness of the organ donor card increased from 101 (44.69%) to 152 (67.25%), while the willingness to donate organs increased from 128 (56.63%) to 151 (66.81%). A community-based cross-sectional study conducted among the adult population in Puducherry revealed that although 72 (28%) individuals possessed adequate knowledge and 148 (60%) exhibited a positive attitude toward organ donation, only six (2.3%) held an organ donor card. This indicates that knowledge and attitude alone are insufficient; effective implementation of organ donation initiatives is crucial for patients with end-stage organ failure [13]. In the present study, there was an improvement in organ donation awareness after the educational intervention, aligning with a study performed among fourth-year medical students in Germany [14]. A study conducted among medical students in South India reported that only 114 (22.4%) students had good knowledge of organ donation, 333 (65.4%) had average knowledge, and 60 (11.74%) had poor knowledge [15]. Some people might have cultural or spiritual views that impede organ donation. These ideas may significantly impact their willingness as organ donors. The study observed that 128 (56.63%) participants perceived religious beliefs as hurdles to organ donation, slightly increasing to 152 (67.25%) after the educational intervention. A study among undergraduate students in Jammu and Kashmir found that 115 (57.7%) declined to donate organs due to religious beliefs [16]. In Saudi Arabia, 1273 (54.7%) respondents were aware of the Islamic Fatwa regarding organ donation, reflecting comparable religious sentiments [17]. The current study found that 118 (52.21%) participants' families were unaware of the value of organ donation, posing a significant barrier. However, following the educational intervention, participants were convinced about the importance of organ donation, as demonstrated by their posttest scores (158; 69.91%). In a 12-year retrospective analysis of brain-dead individuals, similar study results were noted among family members [18]. Bereaved families describe confusion about brain death and organ donation process, emotional and cognitive stress, and decisional conflict, according to a report stated by a qualitative study that combined 34 studies [19]. The outcomes of this study revealed the significant gaps in awareness and barriers to organ donation, particularly among the general population. Although organ donation promotion has been limited in recent years, it is crucial to raise awareness and engage both the public and healthcare professionals. Clinicians and healthcare professionals can play a key role in promoting organ donation through awareness campaigns. Additionally, adopting an opt-out donation policy, as seen in Spain (which has one of the highest organ donation rates) could potentially increase organ donation rates. The term "presumed consent" denotes a legal system that ensures every citizen of a country or state is deemed as a consenting organ donor upon death, unless they actively opt out of participation. However, mixed opinions on the opt-out policy suggest that policymakers may overestimate its effectiveness while underestimating the influence of family decisions [20]. Overall, the study highlights significant post-intervention improvements in awareness and attitudes toward organ donation, emphasizing the life-saving potential of organ donation for individuals with end-stage organ failure.

 Strengths and limitations

The study's major strength lies in its comprehensive examination of awareness and barriers to organ donation across a diverse population of Chengalpattu District, Tamil Nadu. This was achieved through the use of a carefully designed and validated questionnaire. The current study highlights the importance of health education interventions to promote widespread awareness of organ donation. However, the cross-sectional nature of the study and its limited sample size restrict the generalizability of the results to the broader population. Additionally, the overrepresentation of particular groups and the use of convenient sampling introduce selection bias. To address these limitations, future research should employ longitudinal designs, integrate reliable indicators of organ donation behavior, and increase the sample size to encompass a broader range of geographical contexts.

## Conclusions

The present study on health education interventions aimed to inform the general population about the importance of organ donation in India, where road traffic accidents frequently result in brain death, and a lack of awareness potentially limits organ donation opportunities. Even among the well-educated population, there has been a lack of commitment to organ donation, with many not possessing an organ donor card. Although there is a strong inclination to donate, the actual registration rates remain low, suggesting the presence of potential obstacles, such as religious beliefs and insufficient knowledge about organ donation. To address these challenges, targeted educational campaigns involving spiritual leaders and providing accurate data about organ donation are essential. These efforts could significantly improve public awareness and participation. Implementing an opt-out system, where individuals are presumed willing to donate unless they specifically decline, has the potential to substantially increase donor rates. Empirical data from other nations that have adopted opt-out systems indicates a higher prevalence of organ donors compared to opt-in systems. To summarize, increasing organ donation rates in India necessitates a comprehensive strategy that includes focused education, policy reforms, and the resolution of cultural and religious barriers. These initiatives will help bridge the gap between individuals' willingness to donate and their actual registration, ultimately improving health outcomes for patients in need of organ donations.

## References

[1] The Lancet Regional Health-Southeast Asia (2024). Organ transplantation in India: needs a bigger push. Lancet Reg Health Southeast Asia.

[2] NOTTO : National Organ & Tissue Transplant Organisation (2024). NOTTO: National Organ & Tissue Transplant Organization. Cited.

[3] Kupiec-Weglinski JW (2022). Grand challenges in organ transplantation. Front Transplant.

[4] (2024). The global gulf in organ donation rates. https://www.statista.com/chart/27492/deceased-organ-donors-by-country.

[5] Vincent BP, Randhawa G, Cook E (2022). Barriers towards deceased organ donation among Indians living globally: an integrative systematic review using narrative synthesis. BMJ Open.

[6] Tamuli RP, Sarmah S, Saikia B (2019). Organ donation - "attitude and awareness among undergraduates and postgraduates of North-East India". J Family Med Prim Care.

[7] Giri PA, B Y Y, Kamble MG, Solepure AB (2017). Organ donation and transplantation: knowledge and attitude amongst Indian undergraduate medical students. Int J Community Med Public Health.

[8] Darlington D, Anitha FS, Joseph C (2019). Study of knowledge, attitude, and practice of organ donation among medical students in a tertiary care centre in South India. Cureus.

[9] Tanna H, Patel H, Patel P, Patel G, Kumar D (2023). Knowledge and attitude toward organ donation among interns and residents in a tertiary care hospital in Gujarat, India. Cureus.

[10] Chakradhar K, Doshi D, Srikanth Reddy B, Kulkarni S, Padma Reddy M, Sruthi Reddy S (2016). Knowledge, attitude and practice regarding organ donation among Indian dental students. Int J Organ Transplant Med.

[11] Kaur R, Sharma O, Thaman RG (2019). Cross-sectional survey of awareness and attitude towards organ donation and transplantation among students of Punjab. Natl J Integr Res Med.

[12] Bathija G, Ananthesh B G, Bant DD (2017). Study to assess knowledge and attitude towards organ donation among interns and post graduates of a medical college in Karnataka. India Nat J Community Med.

[13] Shah R, Patel A, Ramanuj V, Solanki N (2015). Knowledge and attitudes about organ donation among commerce college students. Nat J Community Med.

[14] Sarveswaran G, Sakthivel MN, Krishnamoorthy Y, Arivarasan Y, Ramakrishnan J (2018). Knowledge, attitude, and practice regarding organ donation among adult population of urban Puducherry, South India. J Educ Health Promot.

[15] Radunz S, Benkö T, Stern S, Saner FH, Paul A, Kaiser GM (2015). Medical students' education on organ donation and its evaluation during six consecutive years: results of a voluntary, anonymous educational intervention study. Eur J Med Res.

[16] Alex P, Kiran KG, Baisil S, Badiger S (2017). Knowledge and attitude regarding organ donation and transplantation among medical students of a medical college in South India. Int J Community Med Public Health.

[17] Khan F, Latif M, Bashir S (2020). Attitude and knowledge toward organ donation among arts and science students. Indian J Forensic Med Toxicol.

[18] Leblebici M (2021). Prevalence and potential correlates of family refusal to organ donation for brain-dead declared patients: a 12-year retrospective screening study. Transplant Proc.

[19] Ralph A, Chapman JR, Gillis J (2014). Family perspectives on deceased organ donation: thematic synthesis of qualitative studies. Am J Transplant.

[20] Molina-Pérez A, Rodríguez-Arias D, Delgado J (2022). Differential impact of opt-in, opt-out policies on deceased organ donation rates: a mixed conceptual and empirical study. BMJ Open.

